# Quantifying the conservation status and abundance trends of wildlife communities with detection–nondetection data

**DOI:** 10.1111/cobi.13934

**Published:** 2022-08-25

**Authors:** Matthew T. Farr, Timothy O'Brien, Charles B. Yackulic, Elise F. Zipkin

**Affiliations:** ^1^ Department of Integrative Biology Michigan State University East Lansing Michigan USA; ^2^ Ecology, Evolution, and Behavior Program Michigan State University East Lansing Michigan USA; ^3^ Global Conservation Program Wildlife Conservation Society Bronx New York USA; ^4^ Southwest Biological Science Center U.S. Geological Survey Flagstaff Arizona USA

**Keywords:** community ecology, demographic rates, dynamic, multispecies modeling, population viability, unmarked modeling, dinámica, ecología comunitaria, modelo multiespecies, modelo sin marca, tasas demográficas, viabilidad poblacional

## Abstract

Effective conservation requires understanding species’ abundance patterns and demographic rates across space and time. Ideally, such knowledge should be available for whole communities because variation in species’ dynamics can elucidate factors leading to biodiversity losses. However, collecting data to simultaneously estimate abundance and demographic rates of communities of species is often prohibitively time intensive and expensive. We developed a multispecies dynamic *N*‐occupancy model to estimate unbiased, community‐wide relative abundance and demographic rates. In this model, detection–nondetection data (e.g., repeated presence–absence surveys) are used to estimate species‐ and community‐level parameters and the effects of environmental factors. To validate our model, we conducted a simulation study to determine how and when such an approach can be valuable and found that our multispecies model outperformed comparable single‐species models in estimating abundance and demographic rates in many cases. Using data from a network of camera traps across tropical equatorial Africa, we then used our model to evaluate the statuses and trends of a forest‐dwelling antelope community. We estimated relative abundance, rates of recruitment (i.e., reproduction and immigration), and apparent survival probabilities for each species’ local population. The antelope community was fairly stable (although 17% of populations [species–park combinations] declined over the study period). Variation in apparent survival was linked more closely to differences among national parks than to individual species’ life histories. The multispecies dynamic *N*‐occupancy model requires only detection–nondetection data to evaluate the population dynamics of multiple sympatric species and can thus be a valuable tool for examining the reasons behind recent biodiversity loss.

## Resumen

La conservación efectiva requiere del entendimiento de los patrones de abundancia de las especies a lo largo del tiempo y el espacio. Sería ideal que dicho conocimiento estuviera disponible para todas las comunidades ya que la variación en la dinámica de las especies puede esclarecer los factores que llevan a la pérdida de la biodiversidad. Sin embargo, la recolección de información para estimar simultáneamente las tasas demográficas y de abundancia de las comunidades de especies con frecuencia es cara y consume tiempo. Desarrollamos un modelo multiespecies dinámico de ocupación‐*N* para estimar la tasa demográfica y de abundancia relativas sin sesgos y en toda la comunidad. En este modelo usamos información de detección‐no detección (p. ej.: censos repetidos de presencia‐ausencia) para estimar los parámetros a nivel comunitario y de especie y los efectos de los factores ambientales. Para validar nuestro modelo, realizamos un estudio de simulación para determinar cómo y cuándo dicha estrategia puede ser valiosa y descubrimos que nuestro modelo multiespecies superó a los modelos comparables de una sola especie en la estimación de las tasas demográficas y de abundancia en muchos casos. Usamos nuestro modelo con datos de una red de cámaras trampa ubicadas a lo largo de África ecuatorial para evaluar los estados y tendencias de una comunidad forestal de antílopes. Estimamos la abundancia relativa, tasa de reclutamiento (es decir, reproducción e inmigración) y las probabilidades de supervivencia aparente para la población local de cada especie. La comunidad de antílopes fue bastante estable (aunque el 17% de las poblaciones [combinaciones especie‐parque] declinaron durante el periodo de estudio). La variación en la supervivencia aparente estuvo vinculada con mayor cercanía a las diferencias entre los parques nacionales que a la historia de vida de cada especie individual. El modelo multiespecies dinámico de ocupación‐*N* requiere solamente información de detección‐no detección para evaluar las dinámicas poblacionales de muchas especies simpátricas y por lo tanto puede ser una herramienta valiosa para examinar las razones detrás de la pérdida reciente de la biodiversidad.

## INTRODUCTION

Information on species’ population dynamics in wildlife communities is often needed to quantify and address threats leading to biodiversity loss (Conde et al., 2019). Estimates of species abundance and demographic rates (e.g., survival, recruitment) tend to rely on marked data (e.g., capture–recapture data), through which individuals are identified and followed via tags, bands, genotypes, or phenotypes (Pollock et al., 1990). Yet, the expensive and labor‐intensive monitoring needed to generate marked data often preclude collection beyond single species at relatively small spatial scales. Though single‐species analyses based on marked data can provide robust inferences, they are often restricted to common or charismatic species (Troudet et al., 2017). Methods to extrapolate single‐species inferences (e.g., from umbrella, keystone, or indicator species) to unmonitored community members may miss important variations among species (Cushman et al., 2010). Accelerating biodiversity loss demands diversified approaches to monitor multiple species simultaneously and whole communities when possible (Nicholson & Possingham, 2006; Zipkin et al., 2020). Community‐wide assessments can provide information about species’ variable responses to environmental factors, including disturbance (Farr et al., 2019).

Although the need to scale up biodiversity assessments to community levels is clear, the required data remain logistically challenging to obtain. Most community‐wide assessments rely on unmarked data (e.g., presence‐only, presence‐absence, detection‐nondetection, count). Unmarked data do not require identification or recapture of individuals and thus can be collected more easily than marked data for community‐wide monitoring. Arguably the most ubiquitous unmarked data type is detection–nondetection data in which the presence or absence of a species is indicated at a given time and place (MacKenzie et al., 2017). A common approach to analyzing detection–nondetection data is occupancy modeling, which makes use of replicate sampling over short time frames to estimate species occurrence patterns while accounting for imperfect detection during sampling (MacKenzie et al., 2002). The advent of multispecies occupancy models (Dorazio & Royle, 2005; Dorazio et al., 2006) has allowed for estimation of community occurrence processes and trends across space and time, driving discoveries in population biology, biodiversity loss, macrosystem processes, and community ecology (Devarajan et al., 2020; Kéry & Schaub, 2012; MacKenzie et al., 2017). Yet without the ability to estimate demographic rates, traditional occupancy models have been restricted in their capacity to allow inference related to changes in population sizes and underlying mechanisms driving trends. Advancements by Royle and Nichols (2003) that link detection probability of a species to local population size created an opportunity to estimate population abundance from detection–nondetection data. Recent development of the “dynamic *N*‐occupancy model” further expanded the use of detection–nondetection data to jointly estimate population abundance and dynamics over time, including demographic rates, for a single species (Rossman et al., 2016). This is done by decomposing annual changes in abundance into apparent survival and populations gains via recruitment (i.e., combination of fecundity and immigration) with the biological process model developed by Dail and Madsen (2011) and the detection model developed by Royle and Nichols (2003).

We expanded the single‐species dynamic *N*‐occupancy modeling framework to a multispecies context that can be used to estimate abundance and demographic rates for communities of related species based only on detection–nondetection data. Our multispecies dynamic *N*‐occupancy model can be used to estimate community‐level responses to environmental covariates and capture species‐specific variation in demographic rates and effects of covariates. By linking species‐specific parameters via community‐level distributions (Dorazio & Royle, 2005; Dorazio et al., 2006), our modeling framework provides abundance and demographic rate estimates for rare and elusive species that otherwise would be unidentifiable with a single‐species approach (Kéry & Royle, 2009; Zipkin et al., 2009, 2010). Scaling the single‐species dynamic *N*‐occupancy model to a multispecies context fills a needed gap in conservation ecology by creating a framework to estimate community‐wide population changes for biodiversity assessments.

We validated our multispecies dynamic *N*‐occupancy model through a simulation study. To demonstrate its utility for conservation, we then applied our model to forest‐dwelling antelope species in tropical equatorial Africa. There is growing concern about human‐induced biodiversity loss in the tropics (Bradshaw et al., 2009). However, tropical communities contain a disproportionate amount of data gaps worldwide (Collen et al., 2008; Meyer et al., 2015) and often only unmarked data collection is feasible (O'Brien, 2008; Tobler et al., 2008). With our multispecies dynamic *N*‐occupancy model, we sought to provide an approach to resolve knowledge gaps in the status and trends of wildlife species and their communities.

## METHODS

We used detection–nondetection data to estimate relative abundance and demographic rates at species and community levels in our multispecies dynamic *N*‐occupancy model. We combined the hierarchical community occupancy modeling framework (Dorazio & Royle, 2005) with the dynamic *N*‐occupancy modeling framework (Rossman et al., 2016), where the latter framework is a dynamic unmarked model (Dail & Madsen, 2011) that assumes an underlying occupancy–abundance correlation (Royle & Nichols, 2003). We used the multispecies dynamic *N*‐occupancy model to estimate latent biological processes (i.e., relative abundance, apparent survival, reproduction, immigration) for individual species and account for imperfect detection during data collection via an observation process component across a series of sites and periods. Species’ biological and observation processes were then linked with a hierarchical statistical structure (i.e., through shared distributions) to estimate community‐ and species‐level parameters. This approach leverages information across species to improve precision of species‐level parameters, especially for species that are observed less frequently due to their rarity or elusiveness (Zipkin et al., 2009).

### Observational process

To estimate latent abundance ($N_{i,j,t}$) of species *i* at a sampling site *j* during year *t*, detection–nondetection data, $y_{i,j,k,t}$, are collected during k=1,2,…K sampling replicates. We assumed that species abundance at a site *j* is closed to changes within year *t*. Thus, the K>1 sampling replicates within a year allow one to estimate the probability that species *i* was detected at site *j* during sampling replicate *k* $\left(y_{i,j,k,t}=1\right)$. We modeled the detection–nondetection data with a Bernoulli process:

(1) $$y_{i,j,k,t}∼\mathrm{Bernoulli}\left(p_{i,j,k,t}\right),$$

where $p_{i,j,k,t}$ is the detection probability of species *i* at site *j* during replicate visit *k* in year *t*.

A nondetection of species *i* can result from 2 separate processes: the species was truly absent at the site (i.e., latent abundance of species *i* at site *j* in year *t* is 0 [$N_{i,j,t}=0$]) or the species was present ($N_{i,j,t}>0$) but no individuals were detected during sampling. Thus, $p_{i,j,k,t}$ can be defined as the probability that at least 1 of the $N_{i,j,t}$ individuals at the site was detected during the *k*th sampling event (Royle & Nichols, 2003):

(2) $$p_{i,j,k,t}=1-{\left(1-\theta_{i,j,k,t}\right)}^{N_{i,j,t}},$$

where $\theta_{i,j,k,t}$ is the detection probability of an individual of species *i* at site *j* during replicate visit *k* in year *t*. If there are no individuals at the site ($N_{i,j,t}=0$), then the detection probability is 0. Likewise, as latent abundance, $N_{i,j,t}$, increases, the overall detection probability, $p_{i,j,k,t}$, of the species also increases because each individual has an independent probability of being detected, $\theta_{i,j,k,t}$. Covariates can be added to $\theta_{i,j,k,t}$ to account for variation in detection by species, site, replicate visit, and year with a logit‐link function:

(3) $$\mathrm{logit}\left(\theta_{i,j,k,t}\right)=\alpha_{0,i}+\alpha_{i}\cdot x_{j,k,t},$$

where $\alpha_{0,i}$ is the intercept for species *i*, or average detection probability of individuals on the logit scale, $\alpha_{i}$ is a vector (1,2,…,V) of parameter coefficients ($\alpha_{1,i},\;\alpha_{2,i},\text{…},\alpha_{V,i}$) for species *i* of corresponding standardized covariates $x_{j,k,t}$ ($x_{1,j,k,t},\;x_{2,j,k,t},\text{…},x_{V,j,k,t}$), which may change by sampling site, replicate visit, and year.

### Biological process

The biological process model focused on estimating $N_{i,j,t}$ for species *i* across all *j* sites in year *t* based on the approach developed by Dail and Madsen (2011). Survey design determines the spatial scale of sampling (Steenweg et al., 2018), and defining the effective sampling area of occupancy surveys can be challenging (Burton et al., 2015). Thus, it is useful to consider inferences on abundance from our model as relative rather than absolute. We assumed that species (relative) abundance changes between years (i.e., from t−1 to *t*) through processes of survival and recruitment. In the first year for which data were available (i.e., t=1), we estimated $N_{i,j,1}$ with a Poisson distribution:

(4) $$N_{i,j,1}∼\mathrm{Poisson}\left(\lambda_{i,j}\right),$$

where $\lambda_{i,j}$ is the expected abundance of species *i* at site *j* in the first year of sampling (Dail & Madsen, 2011). Heterogeneity can be modeled in initial abundance by adding covariates through a log‐link function:

(5) $$\log\left(\lambda_{i,j}\right)=\beta_{0,i}+\beta_{i}\cdot w_{j},$$

where $\beta_{0,i}$ is the intercept (i.e., average initial abundance on the log scale) for species *i* and $\beta_{i}$ is a vector (1,2,…,V) of parameter coefficients ($\beta_{1,i},\beta_{2,i},\text{…},\beta_{V,i}\;$) for standardized covariates $w_{j}\left(w_{1,j},w_{2,j},\cdots ,w_{V,j}\right)$.

In subsequent years (t>1), we assumed that changes to latent abundance of species *i* at site *j* occur via births–deaths and immigration–emigration processes (Dail & Madsen, 2011) and are dependent on the population size during the previous year, t−1. We broke this process into 2 components: $S_{i,j,\;t-1}$, the number of individuals of species *i* that survive from year t−1 to *t* and remain at site *j*, and $\;G_{i,j,t-1}$, the number of new individuals of species *i* that are gained to site *j* via recruitment (reproduction or immigration or both) from year t−1 to *t* (Dail & Madsen, 2011; Rossman et al., 2016). Thus, total abundance in year t>1 is:

(6) $$N_{i,j,t}=S_{i,j,t-1}+G_{i,j,t-1}.$$

We modeled the number of surviving individuals between t−1 and *t* with a binomial distribution:

(7) $$S_{i,j,t-1}∼\mathrm{binomial}\left(N_{i,j,t-1},\;\omega_{i,j,t-1}\right),$$

where $\omega_{i,j,t-1}$ is the apparent survival probability of each individual of species *i* at site *j* between t−1 and *t*. Apparent survival is the product of true survival and site fidelity (i.e., the inverse of permanent emigration). We modeled the number of individuals of species *i* gained into the population at site *j* with a Poisson distribution:

(8) $$G_{i,j,t-1}∼\mathrm{Poisson}\left(\gamma_{i,j,t-1}\right),$$

where $\gamma_{i,j,t-1}$ is the expected number of individuals gained at each site from reproduction and immigration. Provided there are sufficient data, variation in apparent survival probability ($\omega_{i,j,t-1}$) and the expected number of individuals gained to a site ($\gamma_{i,j,t-1}$) can be modeled with covariates that change by site or year or both with a logit‐link function and a log‐link function, respectively.

### Community component

We assumed species in a community share behavioral and ecological similarity but may vary in their life history and responses to environmental stressors (Devarajan et al., 2020). To link the species models at a community level, we assumed that the species‐level parameters (i.e., intercept and effect parameters on either logit‐ or log‐link scales) in both the observation and biological process models are random effects drawn from a parameter‐specific, community‐level distribution shared across all species (Dorazio & Royle, 2005; Dorazio et al., 2006; Zipkin et al., 2009; Zipkin et al., 2010). For example, we assumed the intercept parameter for initial species abundance, $\beta_{0,i}$, comes from a normal distribution:

(9) $$\beta_{0,i}∼\;\mathrm{normal}\left(\mu_{\beta_{0}},\sigma_{\beta_{0}}^{2}\right),$$

with a hypermean $\mu_{\beta_{0}}$ (i.e., representing average expected abundance across all species in the community) and hypervariance $\sigma_{\beta_{0}}^{2}$ (i.e., representing the variation in initial expected abundance across species). The random effects structure allows for information sharing across species within the community, improving parameter identifiability for species with low amounts of data and increasing parameter precision for most other species (Kéry & Royle, 2009; Zipkin et al., 2009). In addition to estimating species‐level biological and observational process parameters, the hierarchical structure of the model also produces estimates of community‐level mean (e.g., $\mu_{\beta_{0}}$) and variance (e.g., $\sigma_{\beta_{0}}^{2}$), which provide useful metrics for summarizing community dynamics.

### Simulation study

To assess our model's performance, we developed a simulation study to measure the accuracy and precision of parameter estimates produced by the multispecies dynamic *N*‐occupancy model. We evaluated our model's performance across a wide range of simulated parameter values. We also compared estimates for individual species parameters generated from the multispecies dynamic *N*‐occupancy model to those produced using equivalent single‐species models (Rossman et al., 2016). For these comparisons, we selected 3 representative species: the species from each simulated community that was most common (i.e., highest latent abundance), rarest (i.e., lowest latent abundance), and most elusive (i.e., fewest detections). Multispecies models are often justified based on their ability to estimate parameters for rare and elusive species (Zipkin et al., 2009); thus, we believe that results from these 3 species types are likely of particular interest to practitioners.

We simulated 1000 communities of 30 species across 10 years at 25 sites and another 1000 communities at 75 sites (following the approach detailed in Rossman et al., 2016). For each of the 2000 communities, we drew hypermean values for initial abundance, gains, and survival from the following, nearly comprehensive, distributions: $\mu_{\lambda }∼\mathrm{uniform}\left(0,1.5\right)$; $\mu_{\gamma }∼\mathrm{uniform}\left(0,1\right)$; and $\mu_{\omega }∼\mathrm{uniform}\left(0,1\right)$. We used the following hyper variances: $\sigma_{\lambda }^{2}=0.25$, $\sigma_{\gamma }^{2}=0.25$, and $\sigma_{\omega }^{2}=0.25$. We opted to keep the variances fixed across simulations to maintain similar structures among communities. We generated species‐specific parameter values via the process detailed in the community component section and used those to simulate latent species abundances following the biological process model. We then simulated the detection–nondetection data assuming that every available site was surveyed on 3 occasions within each year (i.e., during a period of population closure). For each community, we drew a hypermean detection probability from the following distribution $\mu_{\theta }∼\mathrm{uniform}\left(0,1\right)$, assuming a fixed hypervariance of $\sigma_{\theta }^{2}=0.25$, which we used to generate the species‐specific detection probabilities. The data were then simulated following the observation process model.

We estimated parameter values with the multispecies dynamic *N*‐occupancy with a Bayesian framework via NIMBLE and R (de Valpine et al., 2017; R Core Team, 2020; Version 4.0.2) (code is publicly available at https://github.com/zipkinlab/Farr_etal_2022_ConsBiol and https://doi.org/10.5281/zenodo.6513044). We also used the single‐species dynamic *N*‐occupancy model (Rossman et al., 2016) to estimate parameter values for the 3 representative species in each community. All hyperparameters and other fixed‐effect parameters were given vague priors (details in Appendix S1). We ran 3 Markov chain Monte Carlo (MCMC) chains for every data set analyzed, each for 35,000 iterations with a burn‐in of 10,000 and thinning of 25, to provide 3000 samples from the posterior distribution for each parameter. We assessed convergence with the Gelman–Rubin diagnostic (Rhat < 1.1). To assess model performance, we calculated the relative bias (i.e., $\frac{\mathrm{estimated}-\mathrm{true}}{\mathrm{true}}$) for each parameter.

### Case study location and data collection

Our case study focused on a metacommunity of forest‐dwelling antelopes across a network of 6 national parks (Udzungwa Mountains National Park [UDZ], Tanzania; Volcanoes National Park [VNP], Rwanda; Bwindi Impenetrable Forest [BIF], Uganda; Nouabale‐Ndoki National Park [NNNP], Republic of Congo; Korup National Park [KRP], Cameroon; and Nyungwe Forest National Park [NFNP], Rwanda) in the equatorial region of Central and East Africa from 2009 to 2019 (Table 1) (Johnston & Anthony, 2012). We included 12 closely related species (i.e., ecologically similar) (Johnston & Anthony, 2012) (Appendix S2) in our analyses: suni (*Nesotragus moschatus*), bushbuck (*Tragelaphus scriptus*), sitatunga (*Tragelaphus spekii*), and 9 species of duikers (subfamily Cephalophinae) (listed in Table 1). This antelope metacommunity is geographically distributed across Sub‐Saharan Africa and lives in multiple forest types from lowland to alpine (Kingdon, 2015). Each species’ range was limited to a subset of the parks in our study (Table 1). Antelopes face pervasive anthropogenic pressures, including deforestation and poaching (Newing, 2001), and the health, productivity, and persistence of their tropical rainforest habitat are threatened by climate change (Phillips et al., 2009; Sullivan et al., 2020). Recent assessments of this antelope community conflict on species stability (O'Brien et al., 2020), and minimal information on species abundance and demographic rates has prevented conclusive inferences on vulnerability statuses.

**TABLE 1:** Summary information for each national park (NP) included in a multispecies dynamic *N*‐occupancy analysis of the antelope community

Park (abbreviation)	Country	Years of data (*n*)	Species recorded* (*n*)	No. of sampling sites	Total detections across species
Korup NP (KRP)	Cameroon	2011–2015 (5)	5, 8, 12 (3)	60	1100
Nouabale‐Ndoki NP (NNNP)	Republic of Congo	2010–2016 (7)	1, 2, 4, 5, 7, 11, 12 (7)	60	5502
Udzungwa NP (UDZ)	Tanzania	2009–2019 (11)	3, 6, 9, 10 (4)	60	2970
Bwindi NP (BIF)	Uganda	2010–2017 (8)	7, 9, 11, 12 (4)	60	1551
Nyungwe Forest NP (NFNP)	Rwanda	2014–2017 (4)	7, 9, 12 (3)	97	284
Volcanoes NP (VNP)	Rwanda	2014–2016 (3)	7, 9 (2)	60	1432

* Key: 1, *Cephalophus callipygus*; 2, *Cephalophus dorsalis*; 3, *Cephalophus harveyi*; 4, *Cephalophus leucogaster*; 5, *Philantomba monticola*; 6, *Nesotragus moschatus*; 7, *Cephalophus nigrifrons*; 8, *Cephalophus ogilbyi*; 9, *Tragelaphus scriptus*; 10, *Cephalophus spadix*; 11, *Tragelaphus spekii*; 12, *Cephalophus silvicultor*.

We used data from the Tropical Ecology Assessment and Monitoring (TEAM) network to estimate community‐wide abundance and demographic rates (All TEAM data are publicly available at https://www.wildlifeinsights.org/). The TEAM network was developed for monitoring tropical species worldwide with a standardized camera trapping protocol (see TEAM Network, 2011a, 2011b for detailed protocols). Camera traps were deployed in each park, and images were taken whenever an animal triggered a camera's motion sensor. Each park contained camera traps across 60 sites (except NFNP, which had 97 sites) that were sampled once per year for 30 consecutive days. The available years of data varied for parks, ranging from 3 for VNP to 11 for UDZ (Table 1). In postprocessing of images, individual species were identified and aggregated into detection–nondetection histories for 5‐day sampling periods (replicates) and a maximum of 6 replicates per year (based on standard TEAM protocols). In cases where camera traps malfunctioned, we used the amount of time that the camera was functional as a covariate in our observation model to account for variation in detection due to decreased effort. Species were only evaluated at parks within their range; thus, we estimated the abundance and dynamics of 23 populations (i.e., species–park combinations) (Table 1).

### Case study data analyses

We modeled the detection probability of individuals ($\theta_{i,j,r,k,t}$) of each species *i* at site *j* in park *r* during replicate *k* in year *t* with a logit‐link:

(10) $$\mathrm{logit}\left(\theta_{i,j,r,k,t}\right)=\alpha_{0,i}+\alpha_{1}\cdot {\mathrm{days}}_{j,r,k,t},$$

where $\alpha_{0,i}$ is the species‐specific intercept or detection probability of an individual during a sampling replicate when cameras were functional for the average amount of time (4.5 out of 5 days). We added the covariate ${\mathrm{days}}_{j,r,k,t}$ (standardized to a mean of 0 and SD of 1) to incorporate variation in the amount of time that a camera was functional at site *j* at park *r* during replicate *k* in year *t*, an effect that did not vary by species.

For the biological process model, we used a log‐link function to model species’ initial abundances across sites within parks:

(11) $$\log\left(\lambda_{i,r}\right)=\lambda_{0,i}+\varepsilon_{\lambda ,r}.$$

The intercept ($\lambda_{0,i}$) varies by species to account for differences in baseline abundance and a park‐level random effect ($\varepsilon_{\lambda ,r}$) captures variation between parks. We similarly modeled species annual survival probabilities, $\omega_{i,r}$, as

(12) $$\mathrm{logit}\left(\omega_{i,r}\right)=\omega_{0,i}+\varepsilon_{\omega ,r},$$

where $\omega_{0,i}$ is the baseline species‐specific survival probability and $\varepsilon_{\omega ,r}$ is a park‐level random effect. During model development, we explored inclusion of environmental covariates on initial abundance and demographic parameters, but large variations in covariate values between parks prevented meaningful inference and led to overly complex statistical structures (Appendix S2). Further, many of the site‐level (e.g., elevation, temperature, distance to edge) and park‐level covariates were colinear such that it was difficult to determine the important factors influencing species. As such, we settled on including random effects in our estimates of initial abundance and survival to account for species‐specific and community‐level variations between parks without subscribing improper mechanism to estimated differences. We modeled the expected number of individuals gained to a site, $\gamma_{i}$, as

(13) $$\log\left(\gamma_{i}\right)=\;\gamma_{0,i}$$

with only a species‐specific intercept ($\gamma_{0,i}$) because we did not expect residual variation at the park level. There is no evidence for differences in birth rates or sex ratios between parks, and immigration into sites is largely dictated by species’ territorial behavior (such that there is high site fidelity across all species [Kingdon, 2015]), which we assumed did not vary by park.

To link the species models, we drew each species‐specific parameter ($\alpha_{0,i}$, $\lambda_{0,i}$, $\omega_{0,i}$, $\gamma_{0,i}$) from separate community‐level normal distributions with corresponding hypermeans and hypervariances. We used the mean community‐level estimate for apparent annual survival across all parks $\left(\mu_{\omega_{0}}\right)$ in combination with the park‐level random effects on survival ($\varepsilon_{\omega ,r}$) to derive community‐level apparent annual survival for each park (i.e., $\mu_{\omega_{0}}+\varepsilon_{\omega ,r}$). We also derived an index of annual population‐level (i.e., species‐park combination) abundance by summing across sites surveyed in a year. We report average abundance per site (${\hat{N}}_{i,r,t}=\frac{\sum_{j=1}^{J_{r,t}}N_{i,j,r,t}}{J_{r,t}}$) rather than total park abundance to account for variations in sampling effort (i.e., number of sites surveyed per year, $J_{r,t}$ [Table 1]). We calculated annual population growth rates for each population as ${\hat{N}}_{i,r,t}/{\hat{N}}_{i,r,t-1}$ and summarized across years by taking the geometric mean.

We estimated parameters with a Bayesian framework via NIMBLE and R (de Valpine et al., 2017; R Core Team, 2020; Version 4.0.2) (code is publicly available at https://github.com/zipkinlab/Farr_etal_2022_ConsBiol and https://doi.org/10.5281/zenodo.6513044). All hyperparameters and other fixed‐effect parameters were given vague priors (model details in Appendix S3). We ran 5 MCMC chains each for 100,000 iterations with a burn‐in of 75,000 and thinning of 25 providing 5000 samples from the posterior distribution for each parameter. We assessed convergence with the Gelman–Rubin diagnostic (Rhat < 1.1) in addition to visually examining the chains.

## RESULTS

### Simulation study

**FIGURE 1:**
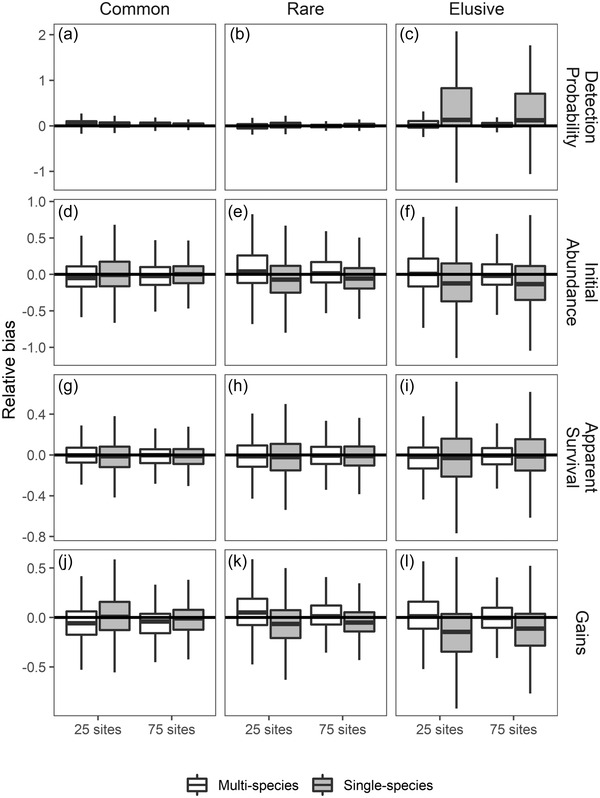
Relative bias ($\frac{\mathrm{estimated}-\mathrm{true}}{\mathrm{true}}$) of (a–c) species‐specific detection probability, (d–f) initial abundance, (g–i) apparent survival, and (j–l) number of individuals gained as estimated using a multispecies dynamic *N*‐occupancy model and comparable single‐species models with simulated data from 25 and 75 sties (common species, highest abundance within the simulated community; rare, species with the lowest abundance; elusive, species with the fewest detections; horizontal line at 0, no bias in estimation; positive values, overestimation; negative values, underestimation; boxes, interquartile range for 1000 simulated communities; center line, median value; whiskers, values within 1.5 times the interquartile range)

The multispecies dynamic *N*‐occupancy model produced estimates with low bias and high precision (Figure 1). Unsurprisingly, accuracy of estimates increased with the number of sites sampled and was also higher for common species and species with high detection probabilities. Our multispecies model outperformed the comparative single‐species models by producing more precise and slightly more accurate parameter estimates in almost all cases for common, rare, and elusive species (Figure 1; comparison of white to gray box plots). The multispecies dynamic *N*‐occupancy model showed the largest improvement in estimates of the biological parameters for elusive species. Detection parameters for the elusive species were only weakly identifiable within single‐species models, which lead to uninformative inferences on biological parameters (Figure 1; right column). However, all parameters for elusive species were estimable within the multispecies model, leading to much more precise inferences on the biological processes for those species with the fewest detections. Although estimates were similar for the common and rare species between both the multispecies and single‐species models when 75 sites were surveyed annually, we observed substantial increases in precision with use of the multispecies model when only 25 sites were available for sampling.

### Case study

**FIGURE 2:**
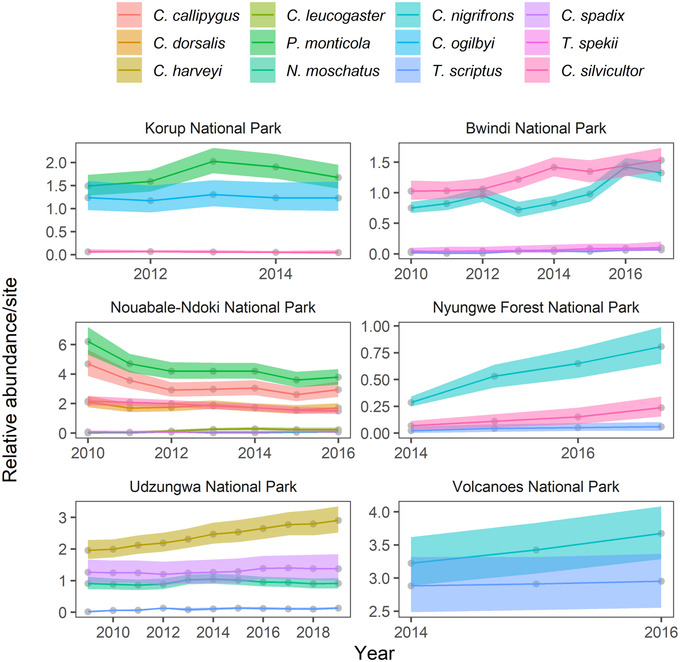
Estimated relative abundance (i.e., abundance per site) of each antelope species at each park across all years that the park was surveyed (points, expected mean abundance; shading, 95% CIs around the mean). Estimates are only shown for parks and years during which sampling occurred and for species that were observed in each park.

Although estimates of relative abundance and population growth revealed that most species within the antelope community were fairly stable over the study period (Figure 2; Appendix S4), 4 of the 23 populations (∼17%) declined in abundance over the 11‐year time frame (95% CI for growth rates was <1). Populations of *Cephalophus callipygus*, *Cephalophus dorsalis*, *Philantomba monticola*, and *Cephalophus silvicultor* had negative growth rates in the Nouabale‐Ndoki National Park (NNNP). Eleven populations had stable population growth rates (95% CI for growth rates overlapped 1) and 8 populations increased in abundance (i.e., 95% CI >1).

**FIGURE 3:**
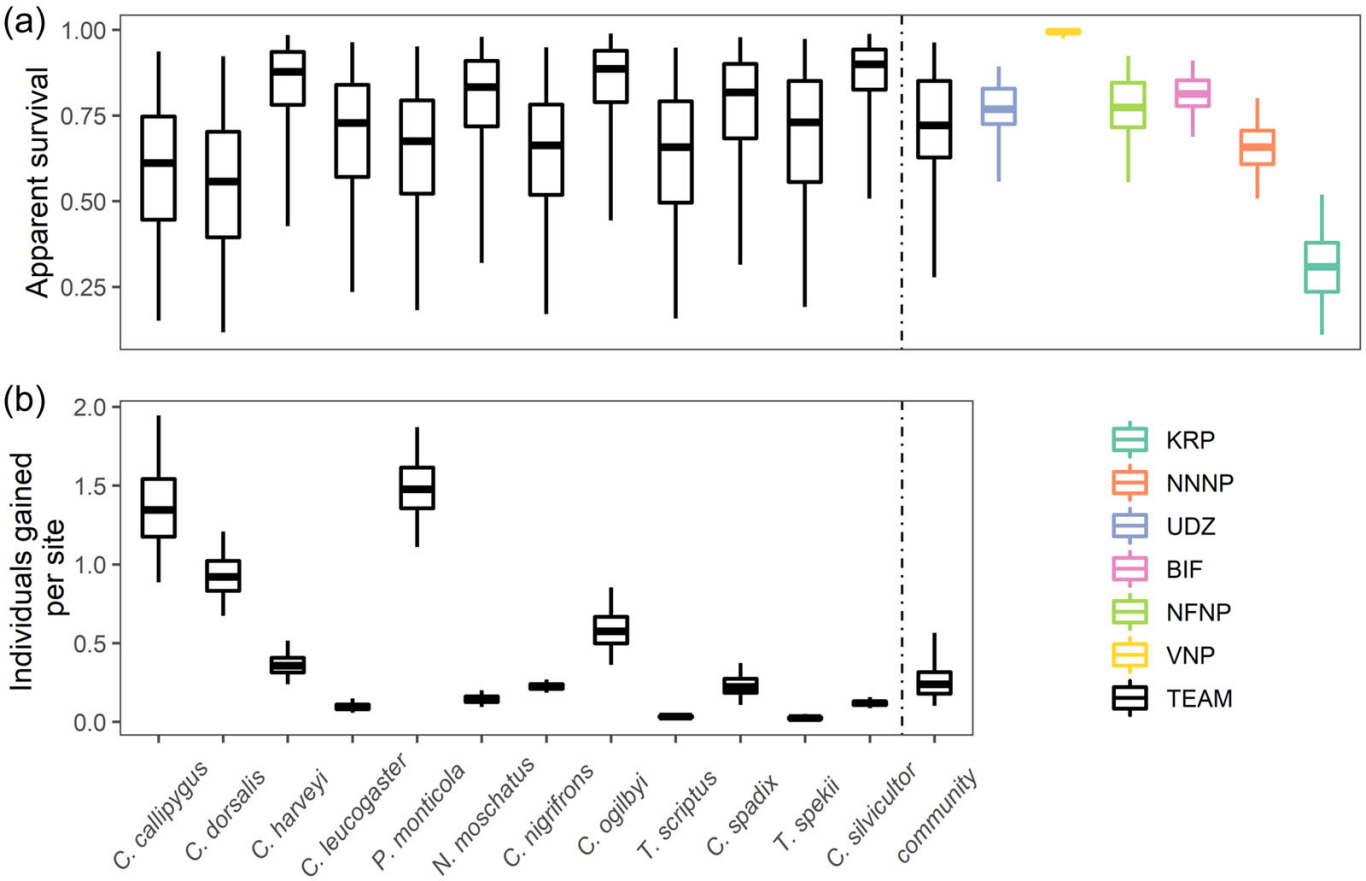
Estimated demographic parameters for individual antelope species and the entire community across parks: (a) annual apparent survival probabilities for each species in the community across the network of parks ($\omega_{0,i}$) (black, left of dashed line), mean survival probability at the community level ($\mu_{\omega_{0}}$) (black, right of dashed line), and mean park‐level survival probabilities for the entire community in the park ($\mu_{\omega_{0}}+\varepsilon_{\omega ,r}$) (colors, right of dashed line); and (b) species‐specific and community‐level annual number of individuals gained ($\gamma_{0,i}$, $\mu_{\gamma_{0}}$) (center lines, expected mean; boxes, 50% CIs; whiskers, 95% CIs; dashed lines, separate species‐specific estimates from the community‐ and park‐level estimates)

Though mean community‐level annual apparent survival ($\mu_{\omega_{0}}$) (Figure 3a) across parks was estimated as 0.72 (95% CI: 0.28, 0.96), there was large variation across species ($\sigma_{\omega_{0}}$ = 0.85 [0.48, 1.52], logit scale) (Appendix S4) and parks ($\sigma_{\varepsilon_{r,\omega }}$ = 2.72 [1.14, 6.50], logit scale) (Appendix S4). The larger variation in survival across parks than across species (i.e., $\sigma_{\varepsilon_{r,\omega }}>\sigma_{\omega_{0}}$) suggests that environmental or anthropogenic factors at the park level may be contributing more to annual survival than species life‐history processes or species‐specific responses to particular environmental factors in a park. Park‐level estimates of survival (Figure 3a) for VNP (0.99 [0.97, 0.99]), BIF (0.81 [0.69, 0.91]), NFNP (0.77 [0.56, 0.92]), and UDZ (0.77 [0.56, 0.89]) were higher than the community‐level average. Despite estimates of stable growth rates of its 3 duiker populations (Figure 2), there was low survival in KRP (0.31 [0.11, 0.52]) (Figure 3a). *Cephalophus silvicultor* had low survival in KRP (0.54 [0.27, 0.72]). Across species, *C. dorsalis* had the lowest average annual apparent survival probability (0.56 [0.12, 0.92]), whereas *C. silvicultor* had the highest (0.90 [0.51, 0.99]) (Figure 3a). The mean number of individuals gained per species annually at sites across parks ($\mu_{\gamma 0}$) was 0.24 (0.10–0.56); *P. monticola* had the highest estimated site‐level recruitment (i.e., sum of immigration and fecundity) (1.48 [1.11–1.87]) and *T. spekii* had the lowest (0.02 [0.01–0.05]) (Figure 3b).

## DISCUSSION

Achieving conservation targets for biodiversity requires quantifiable measures of the status, trends, and dynamics at both species and community levels (Nicholson & Possingham, 2006). The multispecies dynamic *N*‐occupancy model has potential to provide this information for a variety of wildlife communities (provided that certain life‐history assumptions can be met, discussed below). Under conditions explored in the simulation study, our multispecies model generally provided more accurate and precise estimates on individual species parameters as compared to singles‐species analyses. The multispecies dynamic *N*‐occupancy model can also identify average demographic rates of communities while capturing variation among similar species. Use of an extensive continental‐scale camera trapping network in Central and East Africa showed that our multispecies dynamic *N*‐occupancy model estimated stable population growth of antelope species across national parks during the study period; varying abundance and vital rates among species and between populations of the same species; and a lesser effect of species’ life histories on annual apparent survival compared with national park residency. The stronger link between annual apparent survival and national park residency is likely attributable to variation in management enforcement and hunting pressure between parks (Oberosler et al., 2020a, 2020b; O'Brien et al., 2020; Viquerat et al., 2012). The potential of our modeling framework to support biodiversity conservation is underpinned by its ability to quantify demographic rates for multiple species and spatial regions simultaneously with only detection–nondetection data, the most ubiquitous data type for monitoring multiple species.

Contrary to the perception that unmarked data provide limited information relative to marked data, we were able to estimate population abundance and demographic rates for multiple species simultaneously with only detection–nondetection data by merging the hierarchical community (Dorazio & Royle, 2005; Dorazio et al., 2006) and the single‐species dynamic *N*‐occupancy (Rossman et al., 2016) modeling frameworks. Although there is great potential for this approach, multiple limitations may restrict our model's application to certain conditions and communities. In particular, the undefined spatiotemporal sampling scale combined with the structure of the biological process model (i.e., Dail & Madsen's [2011] framework) makes it difficult to estimate absolute values of abundance, apparent survival (i.e., survival and emigration), and number of individuals gained (i.e., births and immigration). Models that estimate abundance based on unmarked data can also have problems with parameter identifiability; thus, checking model fit is advised (Kéry, 2018). Dynamic unmarked models need at least 3–5 years (i.e., time periods) of data for parameters to be identifiable, but simulation results reveal that these models typically do not perform well with < 5–10 years of sampling (Dail & Madsen, 2011; Zipkin et al., 2014). Precision of parameter estimates depends on the number of sites sampled where too few survey locations can lead to inaccuracies (Rossman et al., 2016). In our antelope case study, limited time series for certain populations prevented estimation of site‐level variation within parks or multiscale processes across parks (Appendix S2).

Difficulty with parameter estimation may also occur for species that are rarely observed either because of low abundance or low detection probabilities (Kéry, 2018). Though the multispecies framework theoretically allows for parameter estimation of rare and elusive species (Zipkin et al., 2009), too few observations of a great many species can lead to imprecise or unidentifiable estimates of parameters, especially in the context of a model that aims to estimate demography as well as abundance. However, our simulation study revealed the ability of the multispecies *N*‐occupancy model to estimate parameters for elusive species despite few detections. In some cases, species at the tail end of the community‐level distributions (e.g., very common or very rare) can have their parameter estimates shrunk toward the hypermean values. However, we observed only minimal biases within our simulations (e.g., for gains in common species [Figure 1j]). For some species’ life histories, the basic structure of the multispecies dynamic *N*‐occupancy model may not be feasible. For example, highly mobile and nonterritorial species may violate the geographic closure assumption (i.e., no immigration or emigration out of the site during replicate visits). Other assumptions related to demographic closure (i.e., no births or deaths between replicate visits within a year), independence of sites, independence of individual detections, and species identification may lead to the necessary exclusion of certain taxonomic groups (Royle & Nichols, 2003; Devarajan et al., 2020).

Our results provide an initial demonstration of the multispecies dynamic *N*‐occupancy model and are a prelude to this framework's full potential for conservation science. Further applications of this approach can help explain variation in species statuses and trends by linking environmental drivers (covariates) to demographic rates. Such approaches can elucidate the mechanistic reasons behind biodiversity loss and species declines by partitioning the effects of specific environmental factors on species’ survival and recruitment. The comparatively low cost of collecting unmarked data, such as detection–nondetection data, has made these data types globally available for conservation assessments. When alternative data collection methods are infeasible, our modeling framework can provide community‐wide estimates of abundance and dynamics that are invaluable in informing global conservation priorities.

## Supporting information

Appendix S1 Nimble Model Code for Simulation Study
Appendix S2 Additional case study details
Appendix S3 Nimble model code
Appendix S4 Model output
Table S1. Mean annual population growth rates (i.e., geometric mean) with 95% credible intervals of each population (species‐park combination) during the study period.
Table S2. Mean annual site‐level abundance with 95% credible intervals of each population across the study period (including only the years sampled for each population/park combination).
Table S3. Mean annual survival probability and gains with 95% credible intervals for each species across all parks.
Table S4. Park‐level mean annual survival probability with 95% credible intervals.
