# Supplementary material for: Quantifying the conservation status and abundance trends of wildlife communities with detection–nondetection data
Source: Conserv Biol. 2022 Aug 25;36(6):e13934. doi: 10.1111/cobi.13934 (PMC10086839; doi:10.1111/cobi.13934)
Supplement: Supplementary file 1 — Appendix S1 Nimble Model Code for Simulation Study Appendix S2 Additional case study details Appendix S3 Nimble model code Appendix S4 Model output Table S1. Mean annual population growth rates (i.e., geometric mean) with 95% credible intervals of each population (species‐park combination) during the study period. Table S2. Mean annual site‐level abundance with 95% credible intervals of each population across the study period (including only the years sampled for each population/park combination). Table S3. Mean annual survival probability and gains with 95% credible intervals for each species across all parks. Table S4. Park‐level mean annual survival probability with 95% credible intervals. [file COBI-36-0-s001.pdf]

Supporting Information. Quantifying the conservation status and trends of wildlife communities using detection-nondetection data. Conservation Biology.

Appendix S1

Nimble Model Code for Simulation Study

Nimble code to fit the 'multi-species dynamic *N*-occupancy model' for the simulation study. Please see Github or Zenodo for detailed script to run simulation. Simulation was conducted on Centos 7 Linux cluster using parallel computing.

Definitions of constants

`nSpecies`: number of species (30).
`nSites`: number of sites (25 or 75).
`nYears`: number of years (10).
`nReps`: number of replicates (3).

Model code

```
code <- nimbleCode({

#-Priors-#

# Initial abundance hyperparameters
mu.lambdaL ~ dnorm(0, 0.1) # Community-level intercept log scale
log(mu.lambda) <- mu.lambdaL # Community-level intercept natural scale
tau.lambda ~ dgamma(0.1, 0.1) # Community-level precision
sd.lambda <- 1/sqrt(tau.lambda) # Community-level standard deviation

# Apparent survival hyperparameters
mu.omegaL <- logit(mu.omega) # Community-level intercept logit scale
mu.omega ~ dunif(0, 1) # Community-level intercept probability scale
tau.omega ~ dgamma(0.1, 0.1) # Community-level precision
sd.omega <- 1/sqrt(tau.omega) # Community-level standard deviation

# Gains hyperparameters
mu.gammaL ~ dnorm(0, 0.1) # Community-level intercept log scale
log(mu.gamma) <- mu.gammaL # Community-level intercept natural scale
tau.gamma ~ dgamma(0.1, 0.1) # Community-level precision
sd.gamma <- 1/sqrt(tau.gamma) # Community-level standard deviation

# Detection hyperparameters
mu.rL <- logit(mu.r) # Community-level intercept logit scale
mu.r ~ dunif(0, 1) # Community-level intercept probability scale
tau.r ~ dgamma(0.1, 0.1) # Community-level precision
sd.r <- 1/sqrt(tau.r) # Community-level standard deviation

for(i in 1:nSpecies){

# Species-specific intercept on intial abundance
lambda0[i] ~ dnorm(mu.lambdaL, tau.lambda)
```

```
# Species-specific intercept on apparent survival
omega0[i] ~ dnorm(mu.omegaL, tau.omega)

# Species-specific intercept on gains
gamma0[i] ~ dnorm(mu.gammaL, tau.gamma)

# Species-specific intercept on detection
r0[i] ~ dnorm(mu.r, tau.r)

# Linear predictor for initial abundance
log(lambda[i]) <- lambda0[i]

# Linear predictor for apparent survival
logit(omega[i]) <- omega0[i]

# Linear predictor for gains
log(gamma[i]) <- gamma0[i]

# Linear predictor for detection
logit(r[i]) <- r0[i]

for(j in 1:nSites){

# Initial abundance
N[i,j,1] ~ dpois(lambda[i])

for(t in 2:nYears){

# Apparent survival
S[i,j,t-1] ~ dbin(omega[i], N[i,j,t-1])

# Gains
G[i,j,t-1] ~ dpois(gamma[i])

# Biological process
N[i,j,t] <- S[i,j,t-1] + G[i,j,t-1]

}#end t

for(t in 1:nYears){

# Observation process
p[i,j,t] <- 1 - pow((1 - r[i]), N[i,j,t])

for(k in 1:nReps){

# Observation process
y[i,j,t,k] ~ dbern(p[i,j,t])

}#end k
}#end t
}#end j
}#end i
})
```

Appendix S2

Additional case study details

For the multi-species dynamic *N*-occupancy model case study, we assessed multiple anthropogenic and environmental factors hypothesized to affect the antelope community's abundance and demographic rates. Though the final model did not contain covariates on the biological process, we describe the various covariates that we considered including and an explanation of why we refrained from including covariates in the final model.

Background information

Equatorial Africa has a tropical climate (i.e., wet and humid) and pronounced wet and dry seasons with annual rainfall in parks ranging from 1500 to over 5000 mm (Kingdon 2015, O'Brien et al. 2020). Vegetation in the region is determined by rainfall and elevation with the predominant habitats being lowland and montane rainforest that extend into forest mosaics further from the equator (Kingdon 2015). Elevation varies across parks with the lowest elevation near sea-level in KRP (92 – 463 m) and the highest elevation in VNP (2509 – 3884 m; O'Brien et al. 2020). Parks range in area from 342 – 4000 km^2 and are unfenced with varying human densities (0.5 – 386 humans/km^2) along the edges. Forest fragmentation and resulting refugia during Pleistocene climate change is hypothesized to be responsible for the modern distribution of this community (Johnston & Anthony 2012). In the Anthropocene, humans are likely having the largest influence over the distribution and abundance patterns of this community.

Anthropogenic covariates

Humans may play a role in the spatial distributions and survival of antelope species within our target community as antelopes are often poached as part of the bushmeat trade (Newing 2001). We summarized multiple covariates as a proxy for hunting pressure because direct metrics were not available. We used human population density outside of parks and distance to edge as proxies for anthropogenic disturbance. Because human population density was either high (> 300 humans/km2) or low (< 35 humans/km2) at each park, we specified human density as a binary variable (i.e., 0 for low density [UDZ, NNNP, KRP] and 1 for high density [VNP, BIF, NFNP]). Distance to edge was used as a proxy for hunting pressure and other human disturbance. Distances were recorded from each camera trap site to the nearest accessible edge (e.g., public road, navigable river, park boundary adjacent to human land use).

Environmental covariates

Though previous research on tropical rainforest dwelling antelopes suggest neutral responses to weather conditions (O'Brien et al. 2020), ongoing climate change continues to be a concern for tropical rainforest (Phillips et al. 2009, Sullivan et al. 2020). We characterized local weather (i.e., rainfall) at the site-level to assess the potential influence of climate change on the antelope community. We hypothesized that weather conditions may indirectly affect the population status and trends of antelopes by altering forest productivity and resulting food availability (e.g., foliage, fruit). We used CHIRPS (Climate Hazards group Infrared Precipitation with Stations)

annual precipitation values (mm), which combines satellite and station data to interpolate localized (i.e., 0.05° resolution) rainfall (Funk et al. 2015). Antelope species within this community vary in their diet composition (i.e., percent of foliage, fruit, grass); thus, each species is tied to a different forest types and corresponding elevation range (Kingdon 2015). To capture this variation, we also evaluated a site-level elevation covariate.

Model building with covariates

To interpret multiple covariate effects, we standardized each covariate to have a mean of zero and standard deviation of one. For site-level covariates we standardized across all parks as standardizing within each park prevented meaningful inference. During model development, we first constructed a null model. In later iterations of the model, we attempted to add covariates to abundance, survival, and gains using a forward selection approach (Saunders et al. 2019a, 2019b, Saunders et al. 2021). However, parameter unidentifiability prevented this approach from working as convergence (Rhat < 1.1) for many species-specific effects was not achievable. We hypothesize that large variation in covariate values across parks prevented accurate estimation of effects. For example, precipitation was hypothesized to effect species-specific dynamics; however, large variation between park-level precipitation washed out within park variation. Ideally, population-specific (i.e., interaction between a given covariate, species, and park) effects could account for this issue, but data volumes for many parks were constrained by short temporal extents (Table 1). We also ruled out scaling up site-level covariates (i.e., mean of a park) to the park-level as the low number of parks (i.e., six in total) did not allow covariates to accurately represent (i.e., proxy for true relationship) the anthropogenic or environmental process of interest. Ultimately, we settled on using park-level random effects on initial abundance and apparent survival. The high similarities between sites within a park and the large variations that we observed with covariate values among parks led us to determine that park-level random effects were the most practical solution.

Literature cited

Funk, C., Peterson, P., Landsfeld, M., Pedreros, D., Verdin, J., Shukla, S., … Michaelsen, J. (2015) The climate hazards infrared precipitation with stations–a new environmental record for monitoring extremes. Scientific Data, 2: 150066.

Johnston, A.R. & Anthony, N.M. (2012) A multi-locus species phylogeny of African forest duikers in the subfamily Cephalophinae: evidence for a recent radiation in the Pleistocene. BMC Evolutionary Biology, 12: 120.

Kingdon, J. (2015) The Kingdon field guide to African mammals 2nd edition. London, UK: Bloomsbury Publishing.

Newing, H. (2001) Bushmeat hunting and management: implications of duiker ecology and interspecific competition. Biodiversity and Conservation, 10: 99–118.

O’Brien, T.G., Ahumada, J., Akampurila, E., Beaudrot, L., Boekee, K., Brncic, T., … Strindberg, S. (2020) Camera trapping reveals trends in forest duiker populations in African National Parks. Remote Sensing in Ecology and Conservation, 6: 168–180.

Phillips, O.L., Aragão, L.E.O.C., Lewis, S.L., Fisher, J.B., Lloyd, J., López-González, G., … Torres-Lezama, A. (2009) Drought sensitivity of the Amazon rainforest. Science, 323: 1344–1347.

Saunders, S.P., Ries, L., Neupane, N., Ramírez, M.I., García-Serrano, E., Rendón-Salinas, E., & Zipkin, E. F. (2019a) Multiscale seasonal factors drive the size of winter monarch colonies. Proceedings of the National Academy of Sciences, 116: 8609-8614.

Saunders, S.P., Hall, K.A.L., Hill, N., & Michel, N.L. (2019b) Multiscale effects of wetland availability and matrix composition on wetland breeding birds in Minnesota, USA. The Condor: Ornithological Applications, 121: duz024.

Saunders, S.P., Piper, W., Farr, M.T., Bateman, B.L., Michel, N.L., Westerkam, H., & Wilsey, C.B. (2021) Interrelated impacts of climate and land-use change on a widespread waterbird. Journal of Animal Ecology, 90:1165–1176.

Sullivan, M.J.P., Lewis, S.L., Affum-Baffoe, K., Castilho, C., Costa, F., Cuni Sanchez, A., … Phillips, O.L. (2020) Long-term thermal sensitivity of Earth’s tropical forests. Science, 368: 869–874.

Appendix S3

Nimble model code

Nimble code to fit the 'multi-species dynamic *N*-occupancy model' for the antelope community across the network of TEAM sites within national parks between 2009-2019.

Definitions of constants

`nparks`: number of national parks (6).
`Nspecs`: number of species (12).
`parkS` & `parkE`: indicates the parks where a species was observed (varies by species).
`nsite`: number of sites within a park (varies by park).
`nstart` & `nend`: indicates the years when a park was surveyed (varies by parks).
`nyears`: number of years for projection corresponding to 2030 (22).
`nreps`: number of replicates (6).

Model code

```
model.code <- nimbleCode({

#-Priors-#

# Detection hyperparameters
mu.a0 ~ dunif(0, 1) # Community-level intercept on probability scale
mu.a0L <- logit(mu.a0) # Community-level intercept on logit scale
tau.a0 ~ dgamma(0.1, 0.1) # Community-level precision

# Effect of effort (days sampled)
alpha1 ~ dnorm(0, 0.1)

# Initial abundance hyperparameters
mu.b0 ~ dnorm(0, 0.1) # Community-level intercept on log scale
tau.b0 ~ dgamma(0.1, 0.1) # Community-level precision

# Apparent survival hyperparameters
mu.o0 ~ dunif(0, 1) # Community-level intercept on probability scale
mu.o0L <- logit(mu.o0) # Community-level intercept on logit scale
tau.o0 ~ dgamma(0.1, 0.1) # Community-level precision

# Gains hyperparameters
mu.g0 ~ dnorm(0, 0.1) # Community-level intercept on log scale
tau.g0 ~ dgamma(0.1, 0.1) # Community-level precision

# Precison of park-level random effect on initial abundance
tau.eps.l ~ dgamma(0.1, 0.1)

# Precison of park-level random effect on apparent survival
tau.eps.o ~ dgamma(0.1, 0.1)

for(r in 1:nparks){
```

```
# Park-level random effect on initial abundance
eps.l[r] ~ dnorm(0, tau.eps.l)

# Park-level random effect on apparent survival
eps.o[r] ~ dnorm(0, tau.eps.o)

# Predicted community-level apparent survival for each park
logit(park.surv[r]) <- mu.o0L + eps.o[r]

} # End r

for(i in 1:nspecs){

# Species-specific intercept on detection
alpha0[i] ~ dnorm(mu.a0L, tau.a0)

# Species-specific intercept on initial abundance
beta0[i] ~ dnorm(mu.b0, tau.b0)

# Species-specific intercept on apparent survival
omega0[i] ~ dnorm(mu.o0L, tau.o0)

# Species-specific intercept on gains
gamma0[i] ~ dnorm(mu.g0, tau.g0)

for(r in parkS[i]:parkE[i]){

# Predicted population-level apparent survival
logit(pop.surv[r,i]) <- omega0[i] + eps.o[r]

for(j in 1:nsite[r]){

# Linear predictor for initial abundance
log(lambda[j,r,i]) <- beta0[i] + eps.l[r]

# Initial abundance
N[nstart[r],j,r,i] ~ dpois(lambda[j,r,i])

for(k in 1:nreps){

# Linear predictor for detection probability (in year 1)
logit(r[k,nstart[r],j,r,i]) <- alpha0[i] + alpha1 * days[k,nstart[r],j,i]

# Observation process (in year 1)
y[k,nstart[r],j,r,i] ~ dbern(p[k,nstart[r],j,r,i])

# Site level detection (N-occupancy parameterization) (in year 1)
p[k,nstart[r],j,r,i] <- 1 - pow((1 - r[k,nstart[r],j,r,i]),
N[nstart[r],j,r,i])

} # End k

for(t in (nstart[r]+1):nend[r]){

for(k in 1:nreps){

# Linear predictor for detection probability (in year t+1)
```

```
logit(r[k,t,j,r,i]) <- alpha0[i] + alpha1 * days[k,t,j,i]

# Observation process (in year t+1)
y[k,t,j,r,i] ~ dbern(p[k,t,j,r,i])

# Observation process (in year t+1)
p[k,t,j,r,i] <- 1 - pow((1 - r[k,t,j,r,i]), N[t,j,r,i])

} # End k

# Linear predictor for apparent survival
logit(omega[t-1,j,r,i]) <- omega0[i] + eps.o[r]

# Biological process
N[t,j,r,i] <- S[t-1,j,r,i] + G[t-1,j,r,i]

# Apparent survival
S[t-1,j,r,i] ~ dbin(omega[t-1,j,r,i], N[t-1,j,r,i])

# Gains
G[t-1,j,r,i] ~ dpois(gamma[t-1,j,r,i])

# Linear predictor for gains
log(gamma[t-1,j,r,i]) <- gamma0[i]

} # End t

} # End j

for(t in nstart[r]:nend[r]){

# Population (species-park) abundance per year
Nhat[t,r,i] <- sum(N[t,1:nsite[r],r,i])

} # End t

} # End r

} # End s

})
```

Appendix S4

Model output

Output from the multi-species dynamic *N*-occupancy model. We provide estimates of annual population growth and population abundance during the study period. We also report species-specific, community-level, and park-level demographic rates.

Table S1. Mean annual population growth rates (i.e., geometric mean) with 95% credible intervals of each population (species-park combination) during the study period.

National park	Species	Annual population growth rate
UDZ	*C. harveyi*	1.04 (CI 1.02, 1.06)
UDZ	*N. moschatus*	1.00 (CI 0.98, 1.02)
UDZ	*T. scriptus*	1.19 (CI 1.09, 1.26)
UDZ	*C. spadix*	1.01 (CI 0.98, 1.04)
VNP	*C. nigrifrons*	1.07 (CI 1.03, 1.10)
VNP	*T. scriptus*	1.01 (CI 0.99, 1.03)
NFNP	*C. nigrifrons*	1.41 (CI 1.29, 1.54)
NFNP	*T. scriptus*	1.41 (CI 0.91, 2.08)
NFNP	*C. silvicultor*	1.54 (CI 1.21, 1.94)
BIF	*C. nigrifrons*	1.08 (CI 1.06, 1.11)
BIF	*T. scriptus*	1.12 (CI 0.96, 1.29)
BIF	*T. spekii*	1.16 (CI 0.97, 1.36)
BIF	*C. silvicultor*	1.06 (CI 1.03, 1.09)
NNNP	*C. callipygus*	0.93 (CI 0.90, 0.95)
NNNP	*C. dorsalis*	0.96 (CI 0.93, 1.00)
NNNP	*C. leucogaster*	1.49 (CI 1.33, 1.69)
NNNP	*P. monticola*	0.92 (CI 0.90, 0.95)
NNNP	*C. nigrifrons*	1.07 (CI 0.89, 1.26)
NNNP	*T. spekii*	1.02 (CI 0.81, 1.23)
NNNP	*C. silvicultor*	0.94 (CI 0.91, 0.97)
KRP	*P. monticola*	1.03 (CI 0.98, 1.08)
KRP	*C. ogilbyi*	1.00 (CI 0.93, 1.07)
KRP	*C. silvicultor*	0.92 (CI 0.67, 1.19)

Table S2. Mean annual site-level abundance with 95% credible intervals of each population across the study period (including only the years sampled for each population/park combination).

National park	Species	Year	Site-level abundance
KRP	*P. monticola*	2011	1.49 (CI 1.28, 1.73)
KRP	*P. monticola*	2012	1.59 (CI 1.37, 1.83)
KRP	*P. monticola*	2013	2.02 (CI 1.77, 2.32)
KRP	*P. monticola*	2014	1.90 (CI 1.65, 2.18)
KRP	*P. monticola*	2015	1.68 (CI 1.43, 1.95)
KRP	*C. ogilbyi*	2011	1.24 (CI 0.97, 1.60)
KRP	*C. ogilbyi*	2012	1.17 (CI 0.92, 1.50)
KRP	*C. ogilbyi*	2013	1.30 (CI 1.05, 1.62)
KRP	*C. ogilbyi*	2014	1.23 (CI 0.97, 1.57)
KRP	*C. ogilbyi*	2015	1.23 (CI 0.95, 1.58)
KRP	*C. silvicultor*	2011	0.06 (CI 0.03, 0.12)
KRP	*C. silvicultor*	2012	0.06 (CI 0.05, 0.10)
KRP	*C. silvicultor*	2013	0.06 (CI 0.03, 0.10)
KRP	*C. silvicultor*	2014	0.05 (CI 0.03, 0.08)
KRP	*C. silvicultor*	2015	0.04 (CI 0.02, 0.10)
NNNP	*C. callipygus*	2010	4.69 (CI 3.87, 5.65)
NNNP	*C. callipygus*	2011	3.57 (CI 3.02, 4.23)
NNNP	*C. callipygus*	2012	2.92 (CI 2.47, 3.43)
NNNP	*C. callipygus*	2013	2.97 (CI 2.52, 3.48)
NNNP	*C. callipygus*	2014	3.05 (CI 2.57, 3.58)
NNNP	*C. callipygus*	2015	2.61 (CI 2.17, 3.10)
NNNP	*C. callipygus*	2016	2.94 (CI 2.45, 3.48)
NNNP	*C. dorsalis*	2010	2.10 (CI 1.75, 2.52)
NNNP	*C. dorsalis*	2011	1.69 (CI 1.42, 2.02)
NNNP	*C. dorsalis*	2012	1.75 (CI 1.47, 2.08)
NNNP	*C. dorsalis*	2013	1.90 (CI 1.63, 2.22)
NNNP	*C. dorsalis*	2014	1.71 (CI 1.45, 2.02)
NNNP	*C. dorsalis*	2015	1.57 (CI 1.30, 1.88)
NNNP	*C. dorsalis*	2016	1.69 (CI 1.40, 2.02)
NNNP	*C. leucogaster*	2010	0.01 (CI 0.00, 0.03)
NNNP	*C. leucogaster*	2011	0.04 (CI 0.00, 0.12)
NNNP	*C. leucogaster*	2012	0.14 (CI 0.07, 0.23)
NNNP	*C. leucogaster*	2013	0.25 (CI 0.18, 0.35)
NNNP	*C. leucogaster*	2014	0.28 (CI 0.20, 0.40)
NNNP	*C. leucogaster*	2015	0.23 (CI 0.13, 0.37)
NNNP	*C. leucogaster*	2016	0.23 (CI 0.13, 0.38)
NNNP	*P. monticola*	2010	6.21 (CI 5.32, 7.18)
NNNP	*P. monticola*	2011	4.70 (CI 4.10, 5.37)
NNNP	*P. monticola*	2012	4.19 (CI 3.67, 4.80)

NNNP	*P. monticola*	2013	4.19 (CI 3.68, 4.77)
NNNP	*P. monticola*	2014	4.19 (CI 3.68, 4.75)
NNNP	*P. monticola*	2015	3.59 (CI 3.10, 4.15)
NNNP	*P. monticola*	2016	3.78 (CI 3.28, 4.33)
NNNP	*C. nigrifrons*	2010	0.05 (CI 0.03, 0.08)
NNNP	*C. nigrifrons*	2011	0.01 (CI 0.00, 0.05)
NNNP	*C. nigrifrons*	2012	0.08 (CI 0.07, 0.10)
NNNP	*C. nigrifrons*	2013	0.01 (CI 0.00, 0.05)
NNNP	*C. nigrifrons*	2014	0.02 (CI 0.00, 0.05)
NNNP	*C. nigrifrons*	2015	0.05 (CI 0.02, 0.10)
NNNP	*C. nigrifrons*	2016	0.07 (CI 0.02, 0.15)
NNNP	*T. spekii*	2010	0.07 (CI 0.03, 0.18)
NNNP	*T. spekii*	2011	0.06 (CI 0.00, 0.15)
NNNP	*T. spekii*	2012	0.07 (CI 0.03, 0.15)
NNNP	*T. spekii*	2013	0.05 (CI 0.00, 0.15)
NNNP	*T. spekii*	2014	0.06 (CI 0.00, 0.15)
NNNP	*T. spekii*	2015	0.09 (CI 0.05, 0.18)
NNNP	*T. spekii*	2016	0.08 (CI 0.02, 0.18)
NNNP	*C. silvicultor*	2010	2.17 (CI 1.87, 2.52)
NNNP	*C. silvicultor*	2011	2.08 (CI 1.83, 2.37)
NNNP	*C. silvicultor*	2012	1.99 (CI 1.77, 2.23)
NNNP	*C. silvicultor*	2013	1.84 (CI 1.62, 2.08)
NNNP	*C. silvicultor*	2014	1.72 (CI 1.52, 1.97)
NNNP	*C. silvicultor*	2015	1.54 (CI 1.33, 1.77)
NNNP	*C. silvicultor*	2016	1.50 (CI 1.28, 1.73)
UDZ	*C. harveyi*	2009	1.96 (CI 1.68, 2.28)
UDZ	*C. harveyi*	2010	1.99 (CI 1.73, 2.30)
UDZ	*C. harveyi*	2011	2.12 (CI 1.85, 2.43)
UDZ	*C. harveyi*	2012	2.19 (CI 1.92, 2.52)
UDZ	*C. harveyi*	2013	2.31 (CI 2.03, 2.65)
UDZ	*C. harveyi*	2014	2.47 (CI 2.17, 2.83)
UDZ	*C. harveyi*	2015	2.54 (CI 2.22, 2.92)
UDZ	*C. harveyi*	2016	2.65 (CI 2.32, 3.03)
UDZ	*C. harveyi*	2017	2.77 (CI 2.43, 3.17)
UDZ	*C. harveyi*	2018	2.80 (CI 2.43, 3.23)
UDZ	*C. harveyi*	2019	2.91 (CI 2.52, 3.35)
UDZ	*N. moschatus*	2009	0.91 (CI 0.73, 1.12)
UDZ	*N. moschatus*	2010	0.88 (CI 0.72, 1.07)
UDZ	*N. moschatus*	2011	0.86 (CI 0.70, 1.03)
UDZ	*N. moschatus*	2012	0.89 (CI 0.73, 1.07)
UDZ	*N. moschatus*	2013	1.02 (CI 0.88, 1.18)
UDZ	*N. moschatus*	2014	1.05 (CI 0.90, 1.22)
UDZ	*N. moschatus*	2015	1.02 (CI 0.87, 1.18)

UDZ	*N. moschatus*	2016	0.96 (CI 0.82, 1.12)
UDZ	*N. moschatus*	2017	0.95 (CI 0.80, 1.10)
UDZ	*N. moschatus*	2018	0.90 (CI 0.75, 1.07)
UDZ	*N. moschatus*	2019	0.91 (CI 0.75, 1.08)
UDZ	*T. scriptus*	2009	0.01 (CI 0.00, 0.05)
UDZ	*T. scriptus*	2010	0.06 (CI 0.05, 0.08)
UDZ	*T. scriptus*	2011	0.06 (CI 0.03, 0.10)
UDZ	*T. scriptus*	2012	0.13 (CI 0.12, 0.15)
UDZ	*T. scriptus*	2013	0.08 (CI 0.03, 0.13)
UDZ	*T. scriptus*	2014	0.11 (CI 0.07, 0.15)
UDZ	*T. scriptus*	2015	0.13 (CI 0.10, 0.17)
UDZ	*T. scriptus*	2016	0.12 (CI 0.08, 0.17)
UDZ	*T. scriptus*	2017	0.11 (CI 0.08, 0.15)
UDZ	*T. scriptus*	2018	0.10 (CI 0.07, 0.15)
UDZ	*T. scriptus*	2019	0.13 (CI 0.10, 0.17)
UDZ	*C. spadix*	2009	1.27 (CI 0.93, 1.67)
UDZ	*C. spadix*	2010	1.25 (CI 0.93, 1.63)
UDZ	*C. spadix*	2011	1.25 (CI 0.93, 1.63)
UDZ	*C. spadix*	2012	1.20 (CI 0.85, 1.60)
UDZ	*C. spadix*	2013	1.24 (CI 0.92, 1.63)
UDZ	*C. spadix*	2014	1.26 (CI 0.93, 1.67)
UDZ	*C. spadix*	2015	1.29 (CI 0.93, 1.70)
UDZ	*C. spadix*	2016	1.38 (CI 1.07, 1.78)
UDZ	*C. spadix*	2017	1.40 (CI 1.05, 1.80)
UDZ	*C. spadix*	2018	1.38 (CI 1.02, 1.82)
UDZ	*C. spadix*	2019	1.38 (CI 1.00, 1.83)
BIF	*C. nigrifrons*	2010	0.75 (CI 0.67, 0.85)
BIF	*C. nigrifrons*	2011	0.82 (CI 0.72, 0.93)
BIF	*C. nigrifrons*	2012	0.95 (CI 0.85, 1.08)
BIF	*C. nigrifrons*	2013	0.72 (CI 0.60, 0.85)
BIF	*C. nigrifrons*	2014	0.83 (CI 0.72, 0.95)
BIF	*C. nigrifrons*	2015	0.98 (CI 0.85, 1.12)
BIF	*C. nigrifrons*	2016	1.42 (CI 1.28, 1.57)
BIF	*C. nigrifrons*	2017	1.33 (CI 1.17, 1.50)
BIF	*T. scriptus*	2010	0.02 (CI 0.00, 0.07)
BIF	*T. scriptus*	2011	0.01 (CI 0.00, 0.03)
BIF	*T. scriptus*	2012	0.01 (CI 0.00, 0.03)
BIF	*T. scriptus*	2013	0.04 (CI 0.03, 0.07)
BIF	*T. scriptus*	2014	0.04 (CI 0.03, 0.07)
BIF	*T. scriptus*	2015	0.04 (CI 0.02, 0.07)
BIF	*T. scriptus*	2016	0.06 (CI 0.05, 0.08)
BIF	*T. scriptus*	2017	0.07 (CI 0.05, 0.10)
BIF	*T. spekii*	2010	0.04 (CI 0.02, 0.10)

BIF	*T. spekii*	2011	0.04 (CI 0.02, 0.12)
BIF	*T. spekii*	2012	0.05 (CI 0.02, 0.12)
BIF	*T. spekii*	2013	0.05 (CI 0.02, 0.13)
BIF	*T. spekii*	2014	0.06 (CI 0.02, 0.15)
BIF	*T. spekii*	2015	0.08 (CI 0.05, 0.17)
BIF	*T. spekii*	2016	0.09 (CI 0.05, 0.17)
BIF	*T. spekii*	2017	0.10 (CI 0.05, 0.20)
BIF	*C. silvicultor*	2010	1.02 (CI 0.88, 1.20)
BIF	*C. silvicultor*	2011	1.03 (CI 0.90, 1.18)
BIF	*C. silvicultor*	2012	1.06 (CI 0.92, 1.23)
BIF	*C. silvicultor*	2013	1.22 (CI 1.07, 1.38)
BIF	*C. silvicultor*	2014	1.42 (CI 1.27, 1.58)
BIF	*C. silvicultor*	2015	1.35 (CI 1.17, 1.53)
BIF	*C. silvicultor*	2016	1.44 (CI 1.27, 1.63)
BIF	*C. silvicultor*	2017	1.53 (CI 1.35, 1.73)
NFNP	*C. nigrifrons*	2014	0.29 (CI 0.25, 0.34)
NFNP	*C. nigrifrons*	2015	0.53 (CI 0.42, 0.64)
NFNP	*C. nigrifrons*	2016	0.65 (CI 0.52, 0.79)
NFNP	*C. nigrifrons*	2017	0.81 (CI 0.65, 0.99)
NFNP	*T. scriptus*	2014	0.02 (CI 0.00, 0.06)
NFNP	*T. scriptus*	2015	0.04 (CI 0.01, 0.08)
NFNP	*T. scriptus*	2016	0.05 (CI 0.02, 0.09)
NFNP	*T. scriptus*	2017	0.06 (CI 0.02, 0.10)
NFNP	*C. silvicultor*	2014	0.07 (CI 0.03, 0.11)
NFNP	*C. silvicultor*	2015	0.11 (CI 0.05, 0.18)
NFNP	*C. silvicultor*	2016	0.15 (CI 0.08, 0.24)
NFNP	*C. silvicultor*	2017	0.24 (CI 0.15, 0.34)
VNP	*C. nigrifrons*	2014	3.22 (CI 2.88, 3.62)
VNP	*C. nigrifrons*	2015	3.42 (CI 3.07, 3.83)
VNP	*C. nigrifrons*	2016	3.67 (CI 3.28, 4.08)
VNP	*T. scriptus*	2014	2.88 (CI 2.48, 3.32)
VNP	*T. scriptus*	2015	2.91 (CI 2.52, 3.32)
VNP	*T. scriptus*	2016	2.95 (CI 2.55, 3.37)

Table S3. Mean annual survival probability and gains with 95% credible intervals for each species across all parks.

Species	Annual apparent survival	Gains
C. callipygus	0.61 (CI 0.15, 0.94)	1.34 (CI 0.88, 1.95)
C. dorsalis	0.56 (CI 0.12, 0.92)	0.92 (CI 0.67, 1.21)
C. harveyi	0.88 (CI 0.43, 0.98)	0.36 (CI 0.24, 0.51)
C. leucogaster	0.73 (CI 0.24, 0.96)	0.09 (CI 0.06, 0.15)
P. monticola	0.67 (CI 0.18, 0.95)	1.48 (CI 1.11, 1.87)
N. moschatus	0.83 (CI 0.32, 0.98)	0.14 (CI 0.09, 0.20)
C. nigrifrons	0.66 (CI 0.17, 0.95)	0.22 (CI 0.18, 0.27)
C. ogilbyi	0.89 (CI 0.44, 0.99)	0.57 (CI 0.36, 0.85)
T. scriptus	0.66 (CI 0.16, 0.95)	0.03 (CI 0.02, 0.05)
C. spadix	0.82 (CI 0.31, 0.98)	0.22 (CI 0.11, 0.37)
T. spekii	0.73 (CI 0.19, 0.97)	0.02 (CI 0.01, 0.05)
C. silvicultor	0.90 (CI 0.51, 0.99)	0.12 (CI 0.09, 0.16)
Community-level	0.72 (CI 0.28, 0.96)	0.24 (CI 0.10, 0.56)

Table S4. Park-level mean annual survival probability with 95% credible intervals.

National park	Annual apparent survival
UDZ	0.77 (CI 0.56, 0.89)
VNP	0.99 (CI 0.97, 1.00)
NFNP	0.77 (CI 0.56, 0.92)
BIF	0.81 (CI 0.69, 0.91)
NNNP	0.66 (CI 0.51, 0.80)
KRP	0.31 (CI 0.11, 0.52)
